# Recovery of MRSA and *Clostridium difficile* in an ICU ward

**DOI:** 10.1186/1753-6561-5-S6-P185

**Published:** 2011-06-29

**Authors:** A Ram, AP Gibb, K Templeton, A Holmes, D Swann, CM Taylor

**Affiliations:** 1 Edinburgh Napier University, Edinburgh, UK; 2 Royal Infirmary of Edinburgh, Edinburgh, UK; 3 The University of Edinburgh, Edinburgh, UK

## Introduction / objectives

The role of the hospital environment as a reservoir of infection is poorly understood. Therefore, baseline levels of MRSA (methicillin-resistant *Staphylococcus aureus*) and *Clostridium difficile* contamination on surfaces were determined in an ICU ward. The aim was to determine whether contamination differs at certain times of the day, if recovery of pathogens is influenced by total background contamination and to what extent cleaning affects contamination levels.

## Methods

Sampling occurred in an 18 bed ICU ward on regular days for one month. Surfaces were sampled from patient and non-patient areas, morning and afternoon, to detect total viable counts (TVC), MRSA and *C. difficile* using contact plates and swabs. Multi-locus variable number tandem repeat fingerprinting (MLVF) was performed on selected isolates to determine relationship of MRSA from patients and environment. Statistical analyses were carried out using non-parametric tests.

## Results

Data showed MRSA was recovered from 32% and *C. difficile* recovered from 27% of surfaces. All surfaces tested were contaminated with no significant difference in TVC regardless of time of sampling. MLVF data suggested transfer of MRSA from patient to environment rather than the converse.

## Conclusion

Cleaning was scheduled in the morning however, whether surfaces were re-contaminated after cleaning or whether cleaning was ineffective remains to be established and requires more investigation. Therefore bacteria were detected throughout the surveillance period providing data on baseline levels of MRSA and *C. difficile* on surfaces. Bacterial contamination remains at easily detectable levels despite regular cleaning so ward cleaning protocols may need to be revised.

## Disclosure of interest

None declared.

